# In Vitro Regeneration of Anchote (*Coccinia abyssinica* (Lam.) Cong) Using “Bulla” (*Ensete ventricosum* (Welw.), Cheesman) as an Alternative Gelling Agent

**DOI:** 10.1155/tswj/5655816

**Published:** 2025-06-16

**Authors:** Destaye Shibabaw, Zerihun Demrew Yigezu

**Affiliations:** Department of Plant and Horticultural Sciences, Hawassa University College of Agriculture, Hawassa, Ethiopia

**Keywords:** anchote, cost-effective, enset starch, micropropagation, solidifying agent

## Abstract

In countries that have food security problems like Ethiopia, anchote (*Coccinia abyssinica* (Lam.) Cogn) holds promising potentials for food, feed, and industrial uses. Efficient utilization of such crops through in vitro propagation is limited due to constraints associated with growth medium components. The present study was therefore conducted to evaluate the effectiveness of *Ensete* flour (bulla) as a substitute of agar for micropropagation of anchote using nodal explants. The experiment was conducted using different combinations of bulla and agar as a solidifying agent under completely randomized design (CRD) with factorial arrangement. The earliest and highest shoot initiation was observed when a combination of 75 g/L bulla and 2 g/L agar and 65 g/L bulla and 2.8 g/L agar was used as a solidifying agent, respectively. The highest average shoot number, 8.4 and 6.2, was recorded when 75 g/L bulla and 2 g/L agar and 65 g/L bulla and 2.8 g/L agar were used for micropropagation of red and white accessions, respectively. The maximum leaf counts were registered when the MS medium was supplemented with 75 g/L bulla and 2 g/L agar for red (7.6) and 65 g/L bulla and 2.8 g/L agar for white (7.0) accessions. The number of roots was the highest (12.6 and 12.4) on a growth medium supplemented with 65 g/L bulla and 2.8 g/L agar and 75 g/L bulla and 2 g/L agar for white and red accessions, respectively. The present finding showed that bulla has promising potential to substitute agar in plant tissue growth medium; however, characterization of its starch and identifying the primary active components are required.

## 1. Introduction

*Coccinia abyssinica* (Lam.) Cogn, or anchote, is one the most potentially significant native root tuber crops in Ethiopia. The tuberous crop is a member of the genus *Coccinia*, family Cucurbitaceae, and Order Cucurbitales. There are thirty species in the genus *Coccinia*, and about ten of them exist in Ethiopia [1]. Out of the ten species found in Ethiopia, only *C. abyssinica* is cultivated for human consumptions and known by several colloquial names [2]. It is a crop of great importance in west and southern part of Ethiopia where it is widely grown [3].

The tuberous root has the most starch, is other nutrient-rich part of the plant, and is the main reason for cultivation and recognition of the crop. It can be consumed or used as a reservoir for vegetative development when soil moisture is present [4, 5]. Anchote reported to have 74.93 g of moisture, 3.25 g of protein, and 327 mg of calcium per 100 g of fresh tuber [6]. Of all the major and widely grown root tuber crops, anchote tuber has the highest concentrations of calcium, protein, iron, and vitamin B2 [4]. Reports showed that the edible leaves and fruits of anchote have even more nutrition than the crop's widely utilized tubers [7, 8]. The plant is also known for its medicinal value to treat a variety of ailments, including cancer, gonorrhea, skin eruptions, back pain, and dislocated joints [9]. Although the plant is identified as an important source of nutrient, and known for its medicinal values, its utilization is limited, mainly due to propagation related problems in the plant [10].

Micropropagation is becoming a viable substitute for the large-scale, high-quality, disease-free planting materials. It provides a way to quickly boost valuable accessions and hasten the delivery of enhanced cultivars [11]. Furthermore, healthy seedlings with desired traits that significantly increase productivity are guaranteed via micropropagation [12]. However, there are limitations for wider application of micropropagation, one of which is the essential elements of growth medium. Growth medium gives plant tissue cultures the nourishment and structural support they need to flourish [13]. An affordable and quick to proliferate tissue culture method is important for large-scale mass propagation of anchote [14]. In plant tissue culture, agar with the other elements of the culture medium has been frequently employed as a gelling agent and agar was reported as the most expensive among the growth medium ingredients [15].

One way to lower the overall cost of anchote in in vitro multiplication is to find and optimize inexpensive, readily available starchy gelling agents. Enset (*Ensete ventricosum* (Welw.), Cheesman), one of the commonly grown indigenous crops in Ethiopia, can be considered for this purpose. Enset provides a variety of starchy food products, including kocho, bulla, and amicho [16]. Bulla is a starchy substance with gelling property, insoluble in water, and is processed by squeezing and decanting the liquid followed by open drying. The present work was therefore conducted to evaluate the use of enset (*Ensete ventricosum* (Welw.), Cheesman) flour (bulla) as a substitute of agar in plant growth medium for in vitro propagation of anchote (*Coccinia abyssinica* (Lam.) Cong).

## 2. Materials and Methods

### 2.1. Plant Materials and Consumables

The plant materials (red and white anchote accessions) were obtained from one-year-old previous collections of the plant at Hawassa University, College of Agriculture. Flour of *Ensete ventricosum* (bulla) was purchased from a local farmer, whereas commercially available Murashige and Skoog (MS) basal medium, high-grade plant growth regulators (benzylamimopurine (BAP), indole-3-butyric acid (IBA)), chemicals, reagents, and medium components were obtained from Sigma chemical company, Germany. The plant tissue culture laboratory facilities at Hawassa University, College of Agriculture, were used as required.

### 2.2. Preparation of Explants

Tubers of the two anchote accessions were taken and planted on pots filled with top soil mixed with cattle manure (1:1) under a shade house to be used as a mother plant. Appropriate agronomic managements were given to the seedlings until a single nodal explant size of 3–5 cm was produced and reached their full vegetative stage. Nodal explants were selected and used for initiation, multiplication, and rooting phases.

### 2.3. Experimental Design

The present experiments consisted of red and white anchote accessions and different gelling agents at various concentrations. The experiments were conducted using a completely randomized design (CRD) in factorial combination with five replications (2 × 7 treatments × 5 replications). The use of standard agar alone as a solidifying agent was taken as a control. The SPSS software, Version 29, was employed to design the CRD. The first factor was gelling agent (bulla and agar), and the second factor was anchote accessions (Table 1). The quantitative data were subjected to analysis using SAS statistical software package, Version 9.2 (SAS 2002).

### 2.4. Experimental Procedures

#### 2.4.1. MS Stock Solution

In the present study, the medium stock solutions were prepared separately by weighing the recommended amount of macronutrients, micronutrients, and vitamins according to MS [17] basal medium with minor modifications (Table 1). The solutions were poured into plastic bottles and stored at 4°C until used. The prepared stock solutions were used for a maximum of 1 month.

#### 2.4.2. Preparation of Bulla

Half kilogram of bulla was dissolved in 2 L of water, and the plant debris and fibers were removed with the help of a woven wire mesh (sieve size of 0.2 mm). The contents were then left until the bulla gets separated. The water part was removed, and the precipitated bulla was allowed to dry under open air. The dried bulla was crushed to powder and stored at room temperature until use.

#### 2.4.3. Stock Solution of Plant Growth Regulators

Plant growth regulators such as BAP and IBA were weighed in such a way that every milliliter of a solution contains 1 mg of a given growth regulator and three to four drops of 1 M NaOH was added until the crystals dissolved. The volume was adjusted by double distilled water. Then, the prepared solution was stored at 4°C.

#### 2.4.4. Tissue Culture Medium Preparation

MS basal medium was used for all tissue culture experiments. The medium was prepared by dissolving the required amount of gelling agents (Table 1) and organic and inorganic components in distilled water. pH of the medium was adjusted to 5.8 using either 1 N HCl or 1 N NaOH and heated on a hot plate with continuous stirring using a magnetic stirrer until the content gels dissolved. The contents were dispensed into the culture vessels and autoclaved at a temperature of 121°C for 20 min. The sterilized medium was stored at 4°C to be used in 2 weeks' time.

#### 2.4.5. Surface Sterilization

The nodal explants were washed for 5 min using tap water and liquid detergent along with two drops of Tween-20. The explants were further disinfected under laminar airflow hood with 70% alcohol for 30 s and 1% Clorox bleaches (5.25% NaOCl) for 10 min. After surface disinfection, the explants were rinsed three times with sterilized double distilled water to remove the surface sterilizing agents. In addition, all heat-resistant tools used during the experiment were sterilized at 121°C for 20 min in an autoclave.

#### 2.4.6. Shoot Initiation

The medium used for the initiation stage consisted of MS basal medium supplemented with 1 mg/L of BAP and 30 g/L of sucrose. The sterilized MS basal medium with appropriate treatment was transferred into the culture vessel, and the explants were cultured on the medium. The culture vessels were then kept at 25 ± 2°C for 16 h of light and 8 h of darkness.

#### 2.4.7. Multiplication

After 4 weeks on shoot initiation medium, the explants were moved to multiplication medium. The multiplication medium was made with a different hormone mix but the same procedure as for the start stage. The medium was MS basal medium with 0.5 mg/L of IBA and 1 mg/L of BAP added. Agar was added at the recommended doses when the pH was brought to 5.8. Following the procedure previously mentioned, the medium was autoclaved and then poured into test tube culture vessels. Every 4 weeks, the explants were subcultured and the data was documented.

#### 2.4.8. Rooting

The grown shoots were transferred onto a fresh MS basal medium supplemented with 1 mg/L of IBA and 30 g/L of sucrose, and the shoots were allowed to root. Prior to adding agar, the rooting medium was treated with the prescribed amount of treatment. Twenty milliliters of the medium was added to each test tube culture vessel after it had been autoclaved for 20 min at 121°C. The data was then recorded 4 weeks later.

#### 2.4.9. Acclimatization

For acclimatization, rooted plantlets were taken out of the rooting medium, washed thoroughly under running tap water to remove the adhering agar, and then transplanted on plastic pots containing top soil, compost, and sand at a ratio of 1:2:1. Each pot with plantlet was covered with plastic bags and maintained in greenhouse. The plastic bag was gradually removed after a week and the number of surviving plants in the glasshouse was recorded after 3 weeks.

### 2.5. Cost Analysis

The cost saved by the combined use of bulla and agar as a gelling agent was estimated based on the current market price of both in Ethiopia using the formula given by Mengesha et al. [18]:

(1) $$\begin{array}{c} \text{Cos}t\;saved\;\left(\%\right)=\left(\frac{existing\;gelling\;\cos t-experimental\;gelling\;\cos t}{existing\;gelling\cos t}\right)\times 100, \end{array}$$

where existing and experimental gelling agent cost refers to the cost of a gelling agent when 100% agar and the different combinations of bulla and agar were used, respectively.

### 2.6. Data Collection

Days to shoot and root induction, percentage of rooting and shoot initiation, shoot and leaf numbers per plantlet, shoot length per plantlet (centimeter), internode length, the number of roots per plantlet, root length (centimeter), and survival percentage were recorded.

### 2.7. Data Analysis

All the measured parameters of experiments were subjected to analysis of variance (ANOVA) appropriate to factorial experiments with CRD using SAS software, Version 9.2, and interpretations were made as described by K. A. Gomez and A. A. Gomez [19]. The means were separated by using the least significant difference (LSD) test at the 5% level of significance.

## 3. Results and Discussion

### 3.1. Effect of Bulla as a Gelling Agent for Shoot Initiation of Anchote

#### 3.1.1. Days to Shoot Initiation

The present result showed that days to shoot initiation were significantly (*p* ≤ 0.01) influenced by the interaction of gelling agent and accession and by the main effects. The present finding showed that when 75 g/L of bulla and 2 g/L of agar (red) and 65 g/L of bulla and 2.8 g/L agar (white) with 1 mg/L BAP were used as a gelling agent, the average number of days to shoot initiation was observed to be reduced by half as compared to then 100% agar which was used for both accessions (Table 2). The late shoot initiation with the use of pure bulla as a gelling agent may be associated with high content of carbohydrate in bull that can promote excessive callus formation resulting in delayed shoot initiation. Hassan et al. [20] evaluated different gelling agents for micropropagation of *Philodendron selloum* and showed that the growth and development plantlets were influenced by the nutrient composition of the medium. The biochemical and structural constituent of the medium may affect the diffusion of nutrients, resulting in quantitative variations of shoot induction [21].

#### 3.1.2. Shoot Initiation Percentage

The percentage of shoot initiation showed significant (*p* < 0.01) difference due to the main effects of bulla concentrations and their interaction. The highest shoot initiation percentage (100%) was observed from red and white accessions on growth medium supplemented with 75 g/L bulla + 2 g/L agar and 65 g/L bulla + 2.8 g/L agar, whereas the lowest (48%) was recorded from a medium supplemented with 100 g/L bulla in white accessions (Table 2). Even though there was variation in the response of the two accessions for the concentration of gelling agents, the present finding revealed that the use of bulla as a gelling agent in combination with agar was observed to increase the percentage of shoot initiation under both accessions. Previous report revealed that taro verities were indifferent for shoot induction response for the same growth medium environment [22]. Similarly, Borsai et al. [23] checked eight types of potential gelling agents, and successful shoot regeneration and proliferation of lilac (*Syringa vulgaris* L.) were reported on MS medium supplemented with 4 mg/L BAP.

### 3.2. Effect of Bulla on In Vitro Shoot Multiplication of Anchote

#### 3.2.1. Shoot Number per Plantlet

The shoot number per plantlet was significantly (*p* < 0.01) different due to the main effect of accessions, bulla concentrations, and their interaction (Table 3).

**Figure 1:**
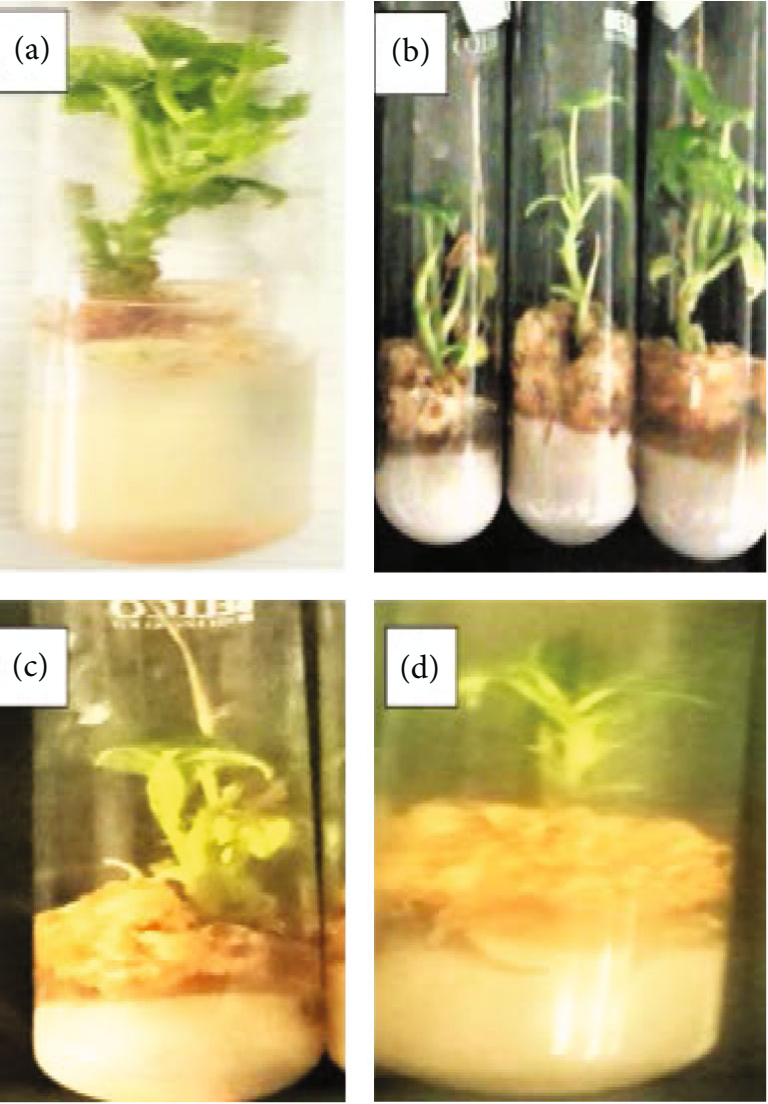
Effects of different combination of bulla and agar on shoot multiplication of anchote accession (a, b: 75 g/L bulla + 2 g/L agar and 65 g/L bulla + 2.8 g/L agar for red and white accessions, respectively; c, d: 100 g/L bulla for both accessions).

The maximum number of shoots (8.4 and 6.2) was recorded on a medium supplemented with 75 g/L bulla with 2 g/L agar and 65 g/L bulla with 2.8 g/L agar for red and white accessions, respectively, whereas, for both accessions, the least number of shoots was recorded from a medium at 100 g/L bulla (Figure 1). In related investigation by Kuria et al. [24], cassava starch has been used as a gelling agent for shoot multiplication of potato. It has been reported that culture medium supplemented with various concentrations of cassava starches was found to be effective gelling agents for inducing and multiplication of shoots. Ullah et al. [25] reported the use of isubgol, cassava, corn, and potato as a gelling agent in plant growth medium, out of which isubgol was the best for the growth of shoots, leaves, and roots in vitro. The present number of shoot per explant was in agreement with the report by Sisay et al. [26].

#### 3.2.2. Leaf Number per Plantlet

**Figure 2:**
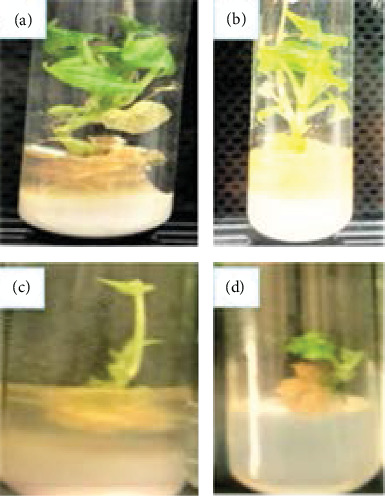
Effects of different concentrations of bulla and agar on leaf number of anchote accessions (a, b: 75 g/L bulla + 2 g/L agar and 65 g/L bulla + 2.8 g/L agar for red and white accessions, respectively; c, d: 100 g/L bulla for both accessions).

The interaction of bulla with accession and the main effect of bulla concentrations highly significantly (*p* < 0.01) influenced leaf numbers during shoot multiplication. However, the leaf number was not significantly affected by the main effect of accessions (Table 4). Red and white accessions gave the maximum mean number of leaves (7.6 and 7.0, respectively) on a medium supplemented with 75 g/L bulla + 2 g/L agar and 65 g/L bulla + agar 2.8 g/L, respectively. On the other hand, for both accessions, the least number of leaves was recorded when bulla was used as a gelling agent alone (Figure 2).

The finding showed that the response of the two accessions to the different concentrations of bulla, in respect of leaf number, was similar to their response in respect of shoot number. In the previous report, Dilebo et al. [27] applied bulla as a gelling agent and reported that 8% (*w*/*v*) enset bulla was ideal and provided a significant number and length of shoots and roots per shoot as compared with 0.6% (*w*/*v*) agar-gelled MS medium.

The reason might be due to its low gel strength, which does not provide enough support for the plant growth. On the other hand, a report on micropropagation of potato (*S. olanum tuberosum* L.) by Venkatasalam et al. [28] showed the least number of leaves obtained at higher concentration of agar. This might be due to the variation in plasticity of plants, and the optimal concentration of bulla to be used as a gelling agent needs to be studied for the different plant species.

#### 3.2.3. Shoot Length per Plantlet

**Figure 3:**
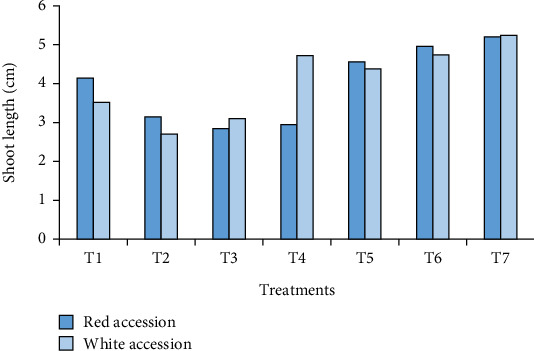
Average shoot length per plantlet as influenced by the use of bulla in combination with agar as a gelling agent.

Shoot length showed a highly significant (*p* < 0.01) difference among bulla with agar concentrations and the interaction of two main factors during in vitro shoot multiplication of anchote. However, the main effect of accessions did not significantly (*p* > 0.05) influence shoot lengths (Table 4). The maximum shoot length per plantlet (5.24 and 5.2 cm) was recorded when agar was used alone (100% agar) in both accessions (Figure 3). The result was in agreement with the finding by Ayenew et al. [29] who used bulla in combination with agar for micropropagation of pineapple (*Ananas comosus* var. *smooth cayenne*).

#### 3.2.4. Internode Length per Plantlet

**Figure 4:**
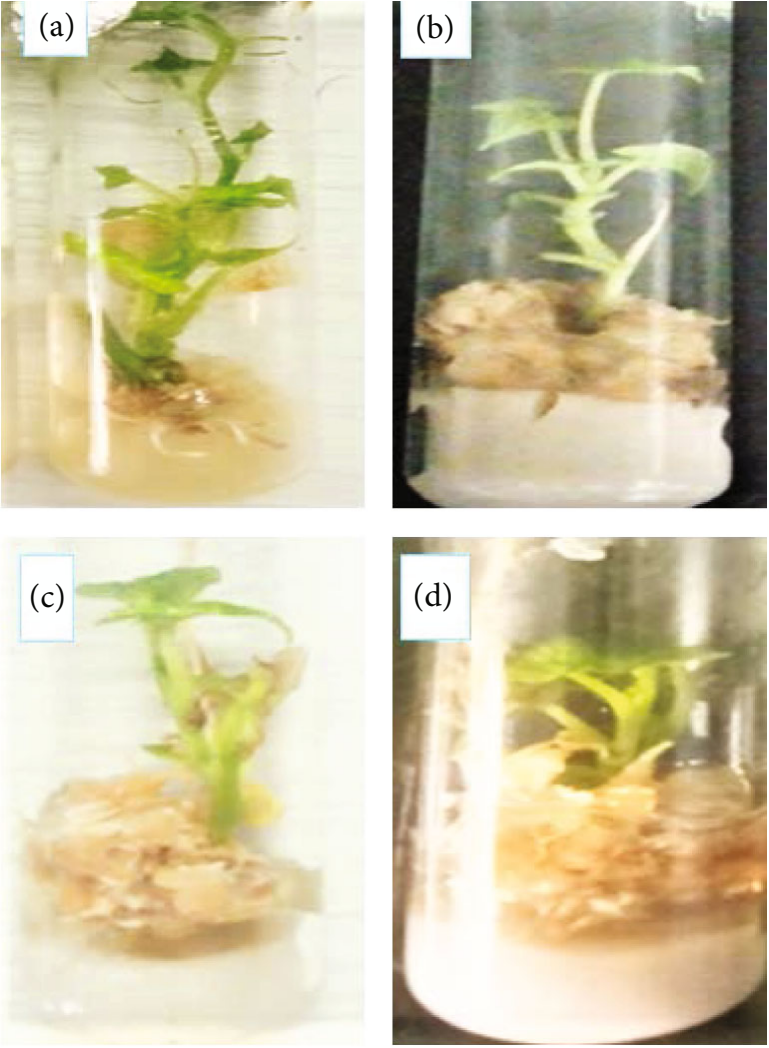
Effects of different concentrations of bulla and agar on internode length of anchote accessions (a, b: 75 g/L bulla + 2 g/L agar and 35 g/L bulla + 5.2 g/L agar for red and white accessions, respectively; c, d: 100 g/L bulla for both accessions).

Red and white accessions gave the maximum mean number of internode length (2.1 and 1.86 cm) on a medium supplemented with 75 g/L bulla + 2 g/L agar and 100 g/L bulla, respectively (Table 4). On the other hand, for both accessions, the least number of internode length (1.32 and 1.24) was recorded on a medium supplemented with 35 g/L bulla + 5.2 g/L agar, respectively (Figure 4). The present finding demonstrates that the internode length of anchote accessions increases with the use of bulla and agar in combination as compared to the use of agar alone as a gelling agent. However, the use of low concentration of bulla as a gelling agent alone showed to decrease the internode length per plantlet. This might be due to that bulla at low concentrations has a high viscosity, which might hinder oxygen supply to the plant tissues, ultimately affecting shoot growth and development. Related investigation by Venkatasalam et al. [28] reported that culture medium supplemented with various concentrations of gelrite starches was found to be effective gelling agents for inducing and multiplication of internode length.

### 3.3. Effect of Bulla on In Vitro Rooting of Anchote Accessions

#### 3.3.1. Days to Root Initiation

The number of days to root initiation was significantly (*p* < 0.01) influenced by interaction of bulla with accession and the main effect of bulla concentrations, and it was also significantly (*p* < 0.05) affected by the main effects of accession (Table 5). The current result showed that root induction occurred early at higher concentrations of agar. The lower gelling ability of bulla as compared to agar might affect the stability of growth medium for the plantlets. However, the high content of starch in bulla is believed to act as a source of nutrients for the developing plantlets, leading to faster growth and development. Choi et al. [30] explored the different growth media and gelling agents for in vitro root regeneration and growth of *P. tinctorium* and suggested that those alternative gelling agents can enhance the rooting abilities in various crops.

#### 3.3.2. Root Initiation Percentage

In the present investigation, root initiation percentage was found to be significantly affected by the main effects of bulla and the interaction of two main factors (Table 5). The highest rooting percentage for red and white accessions was obtained at a combination of higher bulla and lower agar concentration, specifically at 75 g/L bulla + 2 g/L agar (Table 5). This might be due to the higher Zn concentration related to the biosynthesis of indoleacetic acid [31]. It is also observed that when the concentration of bulla decreased, the rooting percentage decreased.

#### 3.3.3. Root Number per Plantlet

**Figure 5:**
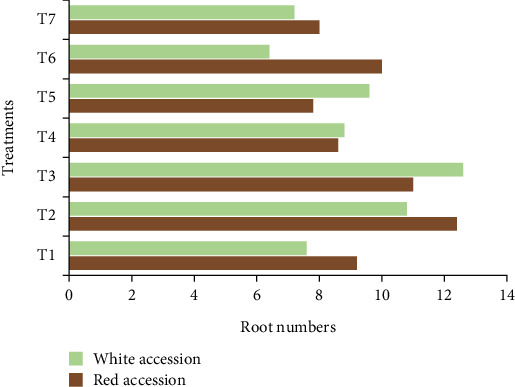
Average root numbers per plantlet as influenced by the interaction of agar and anchote accessions.

The number of roots showed a significant difference (*p* < 0.01) among accessions, bulla concentrations, and the interaction of bulla concentrations and both anchote accessions (Figure 5). The present result indicates that anchote nodal explant cultured on MS medium containing 75 and 65 g/L of bulla as a gelling agent in combination with agar for red and white accessions, respectively, produces significantly higher numbers of roots compared to conventional agar alone. On the other hand, the minimum numbers of roots for red (7.8) and white (6.4) media were obtained from the medium supplemented with 35 g/L bulla + 5.2 g/L agar and 25 g/L bulla + 6 g/L agar for red and white accessions, respectively. The result was in agreement with the findings of Ayalew et al. [8] who reported that 60 g/L bulla + 2 g/L agar gave significantly higher numbers of roots compared to conventional agar for cassava (*Manihot esculenta* Crantz).

#### 3.3.4. Root Length per Plantlet

**Figure 6:**
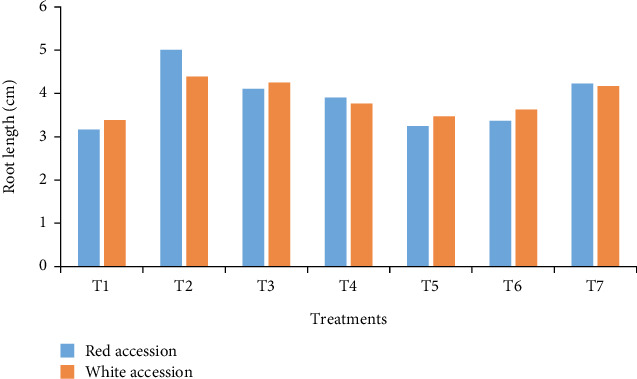
Average root length per plantlet as influenced by the interaction of bulla, agar, and anchote accessions.

The current finding showed that the root length was the highest at a concentration of 75 g/L bulla with 2 g/L agar under MS basal growth medium (Figure 6). It is also revealed that root length was significantly different due to the main effects of bulla concentrations. However, the main effect of accessions did not significantly (*p* > 0.05) influence the root length during in vitro rooting (Figure 6). This might be due to the high carbon content of bulla that enhances root length as compared to conventional agar. Similar results were reported by Ayelign et al. (2012): vanilla propagation by composite of bulla starch with agar as the gelling agent.

### 3.4. Acclimatization of Plantlets

The present investigation showed that plantlets transferred for acclimatization had a survival rate of 84.6% and 75% for red and white accessions, respectively. Plantlets taken from the rooting medium containing 75 g/L bulla in combination with 2 g/L agar and 1 mg/L of IBA showed the best survival rate and growth than the others during acclimatization. This may be due to the better anchoring and absorptive capacities of the vigorous roots produced in that medium. Well-developed root systems are important for the successful development of acclimatized plantlets. The current result also indicated that red accession had the best performance on the rapid survival rate as compared to white accession. In a similar work, Borsai et al. [23] reported that the different gelling agents have a significant impact on the rooting and acclimatization of in vitro–regenerated plants.

### 3.5. Cost Analysis of Gelling Agents

In vitro propagation of anchote has become efficient and promising, but the total cost mostly comes from in vitro medium components that are needed to be reduced by optimizing and/or finding cheap and locally available alternative components. In addition to its unavailability, the commonly used gelling agent, agar, is the most expensive growth medium component in plant micropropagation laboratory. In Ethiopia, bulla starch found to be a cheap and locally accessible gelling agent alternative to agar. Based on the current market price of both agar and bulla in Ethiopia, up to 83.3% of the agar cost in anchote micropropagation could be reduced by using locally available bulla as an alternative gelling agent (Table 6). In a similar fashion, more than 80% plant growth medium cost reduction was reported by the use of isubgol as an alternative gelling agent for in vitro propagation of two African plantain cultivars [32]. The calculated cost in the present work was slightly cheaper than the previous report on the use of bulla and cassava starch as gelling agents for in vitro propagation of vanilla and potato, respectively [18, 24].

## 4. Conclusion

Anchote (*Coccinia abyssinica* (Lam.) accessions) is an important tuber crop in Ethiopia for its food, feed, and medicinal values. However, its propagation is limited to traditional methods due to different constraints like absence of alternative gelling agents. In the present work, bulla (*Ensete ventricosum* (Welw.), Cheesman) was assessed for its use as a solidifying agent under in vitro propagation of two anchote accessions. The result revealed that *Ensete ventricosum*, bulla flour, could be used as a solidifying agent in combination with agar for regeneration of anchote under in vitro. The combination of 75 g/L bulla and 2 g/L agar and 65 g/L bulla and 2.8 g/L agar was the best solidifying agent for in vitro shoot initiation and multiplication of red and white accessions with a corresponding cost reduction of 62.5% and 54.2%, respectively. The observed rooting and shoot initiation in anchote plantlets and the subsequent growth performance might be associated with the addition of carbon by bulla. Even though promising results were obtained with the use of bulla as an alternative gelling agent for anchote in in vitro propagation, further studies need to be conducted to characterize the powder and ascertain the principal active ingredients for wider utilization.

## Figures and Tables

**Table 1:** Treatment combinations of gelling agent for in vitro shoot and root initiation and multiplication of anchote.

**Treatment**	**Bulla (g/L)**	**Agar (g/L)**
T1	100	0
T2	75	2
T3	65	2.8
T4	50	4
T5	35	5.2
T6	25	6
T7	0	8

**Table 2:** Mean value of days to shoot initiation and shoot initiation percentage of anchote as affected by the interaction of bulla, agar, and accessions at 95% level of confidence.

**Accessions**	**Treatment (g/L)**	**Mean days to shoot ** **i** **n** **i** **t** **i** **a** **t** **i** **o** **n** ± **SE**	**Shoot initiation ** **p** **e** **r** **c** **e** **n** **t** **a** **g** **e** ± **SE**
Red	T1	14.4 ± 0.4^bcde^	80 ± 6.3^bcde^
	T2	6.4 ± 0.5^j^	100 ± 0.0^a^
	T3	9.0 ± 0.3^h^	88 ± 8.0^abcd^
	T4	12.8 ± 0.7^f^	84 ± 7.5^abcde^
	T5	13.4 ± 0.5^def^	52 ± 4.9^gh^
	T6	13.8 ± 0.6^cdef^	68 ± 4.9^efg^
	T7	13.2 ± 0.7^e^	56 ± 7.5^fgh^

White	T1	16.2 ± 0.4^a^	48 ± 4.9^h^
	T2	8.2 ± 0.4^hi^	96 ± 4.0^ab^
	T3	7.0 ± 0.5^h^	100 ± 0.0^a^
	T4	10.6 ± 0.4^g^	92 ± 4.9^abc^
	T5	15.0 ± 0.3^abc^	76 ± 7.5^cde^
	T6	15.6 ± 0.5^ab^	80 ± 6.3^bcde^
	T7	14.6 ± 0.5^bcd^	72 ± 4.9^def^

LSD		1.37	16.03

CV		8.88	16.22

*Note:* Treatments with different superscript letters are significantly different in respect of the measured variable at the specified level of probability.

Abbreviations: CV = coefficient of variance, LSD = least significant difference, SE = standard error.

**Table 3:** Mean value of shoot number and internode length of anchote as affected by the interaction of bulla concentrations in combination with agar and accessions.

**Accessions**	**Treatment (g/L)**	**Shoot ** **n** **u** **m** **b** **e** **r** ± **SE**	**Internode ** **l** **e** **n** **g** **t** **h** ± **SE**
Red	T1	3.4 ± 0.0^hij^	1.88 ± 0.0^b^
	T2	8.4 ± 0.1^a^	2.1 ± 0.1^a^
	T3	6.4 ± 0.2^b^	1.72 ± 0.0^cd^
	T4	5.2 ± 0.1^cd^	1.4 ± 0.0^gh^
	T5	4.8 ± 0.2^def^	1.48 ± 0.0^fg^
	T6	4.6 ± 0.1^defg^	1.32 ± 0.0^hi^
	T7	3.8 ± 0.1^ghi^	1.62 ± 0.0^de^

White	T1	2.8 ± 0.0^i^	1.86 ± 0.0^b^
	T2	5.8 ± 0.0^bc^	1.78 ± 0.0^bc^
	T3	6.2 ± 0.0^b^	1.64 ± 0.0^de^
	T4	5.0 ± 0.0^cde^	1.44 ± 0.1^g^
	T5	4.2 ± 0.0^efgh^	1.38 ± 0.1^gh^
	T6	4.0 ± 0.0^fgh^	1.24 ± 0.0^i^
	T7	3.0 ± 0.0^ij^	1.58 ± 0.0^ef^

LSD		0.9	0.12

CV		14.75	5.85

*Note:* Treatments with different superscript letters are significantly different in respect of the measured variable at the specified level of probability.

**Table 4:** Mean square values for shoot multiplication parameters (shoot number, leaf number, shoot length, and internode length) as influenced by accessions, bulla concentrations in combination with agar, and their interactions.

**Source of variation**	**DF**	**Shoot number**	**Leave number**	**Shoot length**	**Internode length**
Accession	1	11.2∗∗	0.51 ns	0.14 ns	0.13∗∗
*B* + *A* + 1 mg/L BAP	6	21.4∗∗	9.31∗∗	7.86 ∗∗	0.59∗∗
Acc∗*B* + *A*	6	1.7∗∗	1.38∗	1.6∗∗	0.03∗∗
Error	56	0.5071	0.6	0.08	0.00879
Total	69				
CV (%)		14.75	13.56	6.89	5.85

*Note: B* = bulla, *A* = agar, Acc = accession. The level of significance was indicated as ns, ∗, and ∗∗, representing not significant at *p* > 0.05, significant at *p* < 0.05, and highly significant at *p* < 0.01, respectively.

Abbreviations: CV = coefficient of variation, DF = degree of freedom.

**Table 5:** Mean days to rooting, length of root, root number, and rooting percentage as affected by accessions, bulla with agar concentration, and their interaction.

**Accessions**	**Treatment (g/L)**	**Mean days to ** **r** **o** **o** **t** **i** **n** **g** ± **SE**	**Root initiation ** **p** **e** **r** **c** **e** **n** **t** **a** **g** **e** ± **SE**
Red	T1	15.4 ± 0.24^bcd^	64 ± 7.5^efgh^
	T2	13.0 ± 0.71^efg^	96 ± 4.0^ab^
	T3	14.0 ± 0.45^def^	76 ± 7.4^cdef^
	T4	14.4 ± 0.51^de^	68 ± 4.89^efg^
	T5	15.2 ± 0.37^cd^	52 ± 4.9^gh^
	T6	16.8 ± 0.37^ab^	48 ± 4.8^h^
	T7	9.0 ± 0.7^h^	100 ± 0.0^a^

White	T1	14.2 ± 0.66^de^	60 ± 6.3^fgh^
	T2	12.0 ± 0.55^g^	100 ± 0.0^a^
	T3	12.6 ± 0.51^fg^	92 ± 4.8^abc^
	T4	15.0 ± 0.6^cd^	88 ± 4.9^abcd^
	T5	18.0 ± 0.32^a^	72 ± 8.0^def^
	T6	16.0 ± 0.45^bc^	64 ± 7.5^efgh^
	T7	14.0 ± 0.71^def^	80 ± 6.3^bcde^

LSD		1.49	16.0

CV		8.28	16.7

*Note:* Treatments with different superscript letters are significantly different in respect of the measured variable at the specified level of probability.

**Table 6:** Cost comparison of gelling agents supplemented with culture medium for anchote in vitro culture.

**Treatment**	**Agar (g/L)**	**Bulla (g/L)**	**Cost of agar (ETB)**	**Cost of bulla (ETB)**	**Cost saved (%)** ^**a**^
T7	8	0	180	0	0
T6	6	25	135	7.5	20.8
T5	5.2	35	117	10.5	29.2
T4	4	50	90	15	41.7
T3	2.8	65	63	19.5	54.2
T2	2	75	45	22.5	62.5
T1	0	100	0	30	83.3

^a^Cost estimated in terms of the current price of gelling agents at the time of this work: 100 g of agar = *€*37.5, which is equivalent to 1*€* = 60 ETB, 100 g of agar = 2250 ETB, and 1 kg of bulla = 300 ETB.

## Data Availability

The data that support the findings of this study are available from the corresponding author upon reasonable request.
